# Supplementary material for: Transcriptomic Changes in Endothelial Cells Triggered by Na,K-ATPase Inhibition: A Search for Upstream Na+i/K+i Sensitive Genes
Source: Int J Mol Sci. 2020 Oct 27;21(21):7992. doi: 10.3390/ijms21217992 (PMC7662270; doi:10.3390/ijms21217992)
Supplement: Supplementary file 1 [file ijms-21-07992-s001.pdf]

Supplementary materials for

Transcriptomic changes in endothelial cells triggered by Na,K-ATPase inhibition: a search for upstream Na^+_i/K^+_i sensitive genes

Elizaveta A. Klimanova [1*], Svetlana V. Sidorenko [1], Polina A. Abramicheva [1], Artem M. Tverskoi [1], Sergei N. Orlov [1,†], and Olga D. Lopina [1]

[1] Faculty of Biology, Lomonosov Moscow State University, Moscow, Russia, 119234; sidorenko.svetlana.v@yandex.ru (S.S.), abramicheva.polina@gmail.com (P.A.), tverskoiam@gmail.com (A.T.), od_lopina@mail.ru (O.L.)

* Correspondence: klimanova.ea@yandex.ru; Tel.: +7-(495)-939-4434

† Deceased

Table S1. Time-dependent actions of Na,K-ATPase inhibition on Na^+_i/K^+_i-content and $[Na^+]_i/[K^+]_i$-ratio.

hr	Na^+_i (nmol/mg prot)			K^+_i (nmol/mg prot)		
	control	*ouabain*	*K⁺-free*	*control*	*ouabain*	*K⁺-free*
**0.5**	42±5	79±6	99±8	440±66	309±28	218±29
**1**	52±8	127±12	172±23	504±15	292±26	115±9
**2**	41±3	152±12	182±32	502±21	171±14	23±12
**6**	51±3	234±28	237±17	444±20	23±2	18±4

hr	$[Na^+]_i/[K^+]_i$-ratio		
	*control-r.un(**lg**)*	*ouabain*	*K⁺-free*
**0.5**	0.095-1.00(**0**)	0.256-2.69(**0.430**)	0.454-4.78(**0.680**)
**1**	0.103-1.08(**0.033**)	0.435-4.58(**0.681**)	1.50-15.8(**1.20**)
**2**	0.082-0.86(**-0.066**)	0.889-9.35((**0.971**)	7.91-83.3(**1.92**)
**6**	0.115-1.21(**0.083**)	10.1-106(**2.03**)	13.2-139(**2.14**)

Table S2. Common differentially expressed genes triggered by Na,K-ATPase inhibition by 3 μM ouabain or K^+-free medium in 2 h.

**Gene Symbol**	**3 μM ouabain**		**K⁺-free medium**	
	FC	P-value	FC	P-value
RNU6-1263P	2,72571	2,80E-08	3,76	1,50E-11
RN7SL600P	2,71287	8,26E-06	8,81	9,24E-13
C9orf131	2,52044	6,93E-06	3,28	1,35E-08
RN7SL473P	2,44738	1,03E-07	7,58	1,26E-15
CD274	2,30808	1,06E-10	4,3	3,40E-17

Gene Symbol	3 μM ouabain		K^+-free medium	
	FC	P-value	FC	P-value
IL1A	2,20675	8,31E-09	5,83	1,18E-16
RPL21P44	2,20209	1,12E-07	2,2	1,18E-08
SPRY4	2,00014	4,45E-09	2,39	5,86E-12
CXCL8	1,96913	6,99E-07	2,42	6,35E-10
STPG4	1,95932	2,05E-07	3,34	3,96E-13
KCNJ2	1,94119	1,85E-06	2,03	7,44E-08
SLC25A25	1,92091	1,77E-09	3,39	4,70E-16
IL1RL1	1,83287	6,40E-10	4,9	2,32E-19
RN7SKP208	1,79604	5,44E-06	-2,17	6,37E-09
SNORD13E	1,78566	4,64E-05	2,37	1,10E-08
OR7E115P	1,76619	5,90E-03	2,28	1,58E-05
GEM	1,75946	8,04E-06	3,72	2,42E-13
TSC22D2	1,74905	3,39E-10	4,53	5,64E-20
AL356356.2	1,74624	5,49E-05	2,1	9,95E-08
SLF1	1,73348	6,43E-03	2,45	3,29E-06
MTFR2	1,66211	4,25E-04	2,43	1,45E-08
GPR75	1,64897	5,39E-05	1,75	1,65E-06
AP1AR	1,64452	1,52E-02	2,62	1,13E-06
RNU6-1092P	1,61745	2,64E-02	2,14	5,74E-05
SNAPC1	1,60158	3,49E-07	2,73	2,01E-14
POLG2	1,57468	7,97E-06	2,14	1,18E-10
CSRNP1	1,56232	3,48E-05	3,07	3,96E-13

Gene Symbol	3 μM ouabain		K^+-free medium	
	FC	P-value	FC	P-value
RND3	1,53824	1,63E-10	2,38	6,72E-18
KITLG	1,53196	5,75E-05	5,36	1,11E-16
RN7SL849P	1,52751	6,66E-03	6,33	6,76E-14
CHAC1	1,52751	9,97E-04	1,53	1,88E-04
RNU1-87P	1,52316	3,31E-03	2,04	5,84E-07
ADAMTSL4	1,51901	1,50E-03	2,97	7,10E-11
TIFA	1,51136	9,49E-07	2,19	7,56E-13
RN7SKP150	1,50144	3,29E-02	2,1	1,37E-05
RNU6-1231P	-1,51381	5,28E-04	-1,86	2,82E-07
INKA1	-1,52765	7,06E-04	-2,05	5,18E-08
RNPC3	-1,54386	8,28E-03	-2,49	9,23E-08
ANXA2R	-1,50183	1,20E-03	-1,68	1,02E-05
ANKRD49	-1,56547	7,10E-03	-1,69	3,06E-04
ALX1	-1,56564	1,74E-03	-1,87	4,34E-06
HOXA9	-1,60854	1,18E-08	-2,36	1,19E-14
ZSCAN16	-1,60994	7,42E-04	-2,63	2,52E-09
HMOX1	-1,62669	2,25E-04	-1,7	1,24E-05
GIMAP7	-1,65804	1,00E-03	-2,19	4,06E-07
SLC15A4	-1,69082	1,80E-03	-3,09	3,77E-09
DEPP1	-1,69456	8,84E-06	-4,99	9,57E-16
TRIM52	-1,22752	3,75E-01	-2,14	4,76E-06
SNORD96A	-1,75891	1,96E-03	-2,18	5,98E-06

**Gene Symbol**	**3 μM ouabain**		**K^+-free medium**	
	FC	P-value	FC	P-value
AC002350.1	-1,78216	3,43E-03	-3,16	4,24E-08
FLJ35409	-2,1326	2,02E-03	-2,85	5,98E-06
LOC100130713	-2,14445	9,38E-07	-3,49	1,12E-11
SNORD52	-2,19944	1,72E-06	-3,96	7,64E-12

Table S3. Prediction of G-quadruplexes within early response genes' DNA.

Sequence definition	NCBI Reference Sequence	Begining of G4 sequence (first nucleotide=0)	End of G4 sequence	Length	Given sequence (+ strand)	G4-forming sequence	G4HScore
C7ORF53	NC_000007.14	5835	5858	23	GGGTGTGGTGGAGGGGGAGGGGG	GGGTGTGGTGGAGGGGGAGGGGG	2.522
C7ORF53	NC_000007.14	6220	6244	24	GGGTTGGAGAAAGGGGGTGGGGGG	GGGTTGGAGAAAGGGGGTGGGGGG	2.417
C7ORF53	NC_000007.14	9144	9167	23	GGGTTGGGACTGGGAGGGAGGGG	GGGTTGGGACTGGGAGGGAGGGG	2.217
C7ORF53	NC_000007.14	11365	11385	20	GGGGTGGGCGGCTAGCAGGG	GGGGTGGGCGGCTAGCAGGG	1.8
EGR1	NC_000005.10	822	852	30	GGAGCTGCGTGGGTGGGTGGAGGG GGAGGG	GGAGCTGCGTGGGTGGGTGGAGGG GGAGGG	1.867
FOS	NC_000014.9	341	368	27	GGGGCCGGGGGCTTGGGGTCGCGG AGG	GGGGCCGGGGGCTTGGGGTCGCGG AGG	2
FOS	NC_000014.9	705	745	40	GGAGGAGGGATGAGGGAGGAGGGT GCAGCGGGCGGGTGTG	GGAGGAGGGATGAGGGAGGAGGGT GCAGCGGGCGGGTGTG	1.475
FOS	NC_000014.9	1405	1427	22	GGGAATGTGGGGGCTGGGTGGG	GGGAATGTGGGGGCTGGGTGGG	2.136
FOS	NC_000014.9	2755	2778	23	GTGAGGGGGCAGGGAAGGGGAGG	GTGAGGGGGCAGGGAAGGGGAGG	2.174
FOSB	NC_000019.10	912	970	58	GGGAACGCGGCAGGGAGGGTAGGC TTTGGGGCGAGGTGGGGGTGGGGTG GGTAATGCGTAATGCG	GGGAACGCGGCAGGGAGGGTAGGC TTTGGGGCGAGGTGGGGGTGGGGTG GGTAATGCGTAATGCG	1.523
FOSB	NC_000019.10	2242	2265	23	GGGGTGGGGGTGGGGTGTTGTGG	GGGGTGGGGGTGGGGTGTTGTGG	2.522
FOSB	NC_000019.10	3315	3347	32	GGCCCTGGGGTGGGAGAGGGGATG TTGAGGGG	GGCCCTGGGGTGGGAGAGGGGATG TTGAGGGG	1.719
FOSB	NC_000019.10	3557	3587	30	GGGGCCACATGGGGCCCTTGAGGA GAGGGG	GGGGCCACATGGGGCCCTTGAGGA GAGGGG	1.333

Sequence definition	NCBI Reference Sequence	Begining of G4 sequence (first nucleotide=0)	End of G4 sequence	Length	Given sequence (+ strand)	G4-forming sequence	G4HScore
FOSB	NC_000019.10	3910	3930	20	GGGAGGTAGAGAGGGAGGGG	GGGAGGTAGAGAGGGAGGGG	2
FOSB	NC_000019.10	4683	4706	23	GGGGCCCGGGCCGGGCCCGCTGG	GGGGCCCGGGCCGGGCCCGCTGG	0.696
FOSB	NC_000019.10	5243	5278	35	GGTGACTTTGGGGACAGGGGGTGG GAAGGGGATGG	GGTGACTTTGGGGACAGGGGGTGG GAAGGGGATGG	1.94
FOSB	NC_000019.10	5323	5348	25	GGGGATGGGTGGGGAGGGGGGCGG G	GGGGATGGGTGGGGAGGGGGGCGG G	2.92
FOSB	NC_000019.10	5376	5418	42	GAGGATTAAGGGACGGGGGTGGGA GGTAGGCTGTGGGGTGGG	GAGGATTAAGGGACGGGGGTGGGA GGTAGGCTGTGGGGTGGG	1.786
MLF1	NC_000003.12	163	209	46	GGGAGCCAGGAGTCCGGGGTCGCG CGGGAATTAAGGTATCGAGGGG	GGGAGCCAGGAGTCCGGGGTCGCG CGGGAATTAAGGTATCGAGGGG	1.109
MLF1	NC_000003.12	409	437	28	GGGAAGTTTGGGGGCGTGAGGTGTG GGG	GGGAAGTTTGGGGGCGTGAGGTGTG GGG	1.857
MLF1	NC_000003.12	479	506	27	GGGAGGTGGGGGGAGGAGAATTGA GGG	GGGAGGTGGGGGGAGGAGAATTGA GGG	1.926
MLF1	NC_000003.12	530	710	180	GGGCGTGAGGTGAGGGAAGGGTTT GGGAGCATGAGGTGGGAGGGAGGA GGGTTGAGGGCGTGAGGTGAGGGG AAGGGTTGAGGGTGTGAGGTGAGG GGAAGGGTTGAGGGCGTGAGGTGA GGGGTTGAGGGCGTGAGGTGAGGG GAAGGGTTGAGGGCGTGAGGTGGG GGAAGGG	GGGCGTGAGGTGAGGGAAGGGTTT GGGAGCATGAGGTGGGAGGGAGGA GGGTTGAGGGCGTGAGGTGAGGGG AAGGGTTGAGGGTGTGAGGTGAGG GGAAGGGTTGAGGGCGTGAGGTGA GGGGGGTTGAGGGCGTGAGGTGAG GGGAAGGGTTGAGGGCGTGAGGTG GGGGAAGGG	1.594

Sequence definition	NCBI Reference Sequence	Begining of G4 sequence (first nucleotide=0)	End of G4 sequence	Length	Given sequence (+ strand)	G4-forming sequence	G4HScore
MLF1	NC_000003.12	747	835	88	GTGAGGTGGGGGGAGGGTTTGGGA GCGTGGGGTGGGAGGAGGAGGGTT TGGGGGAGTAAGGTGGAGGGGGGA GGGTTGAGGGTGTGAG	GTGAGGTGGGGGGAGGGTTTGGGA GCGTGGGGTGGGAGGAGGAGGGTT TGGGGGAGTAAGGTGGAGGGGGGA GGGTTGAGGGTGTGAG	1.886
MLF1	NC_000003.12	876	927	51	GGGTTGAGGGCGTGAGGTGGGGGG AGGGTTTGGGAGCATGAGGTGTGGG GG	GGGTTGAGGGCGTGAGGTGGGGGG AGGGTTTGGGAGCATGAGGTGTGGG GG	1.804
MLF1	NC_000003.12	986	1039	53	GGGAGCATGAGGTGTGGGGGAGGG TTGAGGGTGTGAGGTGAGGGGAAG GAGGG	GGGAGCATGAGGTGTGGGGGAGGG TTGAGGGTGTGAGGTGAGGGGAAG GAGGG	1.698
MLF1	NC_000003.12	1048	1089	41	GCGAGGTGGGGGGAGGAGGGTTGA GGGCGTGAGGTGGGGGG	GCGAGGTGGGGGGAGGAGGGTTGA GGGCGTGAGGTGGGGGG	1.976
MLF1	NC_000003.12	1133	1180	47	GTGCAGGGACGAGGGTTGAGGGCG TGAGTTGGTGGGGAGGGTTTGGG	GTGCAGGGACGAGGGTTGAGGGCG TGAGTTGGTGGGGAGGGTTTGGG	1.468
MLF1	NC_000003.12	1305	1369	64	GGGGGCATGAGGTGTGGGAGGAGG GTTGGGGAGAAGGGTTGAGGGCGT GAGTTGGTGGGAAGGG	GGGGGCATGAGGTGTGGGAGGAGG GTTGGGGAGAAGGGTTGAGGGCGT GAGTTGGTGGGAAGGG	1.672
MLF1	NC_000003.12	1385	1416	31	GGGAGGAGGGTTGAGGGCGTGAGG TAGGGGG	GGGAGGAGGGTTGAGGGCGTGAGG TAGGGGG	1.839

Sequence definition	NCBI Reference Sequence	Begining of G4 sequence (first nucleotide=0)	End of G4 sequence	Length	Given sequence (+ strand)	G4-forming sequence	G4HScore
MLF1	NC_000003.12	1427	1559	132	GGGTGAGAGGTAGTGGGACGAGGGAGGGTTGGGGCGTGAGGTGGAGGGAGGAGGGTTTGGGGGCATGAGGTGTGGGGAAGAGGGTTTAGGGGCATGAGGTGTGGGGGTAGGAGGGTTGAGGGTGTAGGTGG	GGGTGAGAGGTAGTGGGACGAGGGAGGGTTGGGGCGTGAGGTGGAGGGAGGAGGGTTTGGGGGCATGAGGTGTGGGGAAGAGGGTTTAGGGGCATGAGGTGTGGGGGTAGGAGGGTTGAGGGTGTGAGGTGG	1.629
MLF1	NC_000003.12	1575	1613	38	GGCGTGCAGTGGGGGGAGGAGGGTTTGGGGGCGTGAGG	GGCGTGCAGTGGGGGGAGGAGGGTTTGGGGGCGTGAGG	1.763
MLF1	NC_000003.12	2170	2225	55	GGGGCGGGGCGGGGGGAGGGGGGCGGGAGGAGGGAGGAGGGAGGAGGGCGGCGGGGGGGGGGGGCGGCGGGGGGGGGGGGTGTGTGTG	GGGGCGGGGCGGGGGGAGGGGGGCGGGAGGAGGGAGGAGGGAGGAGGGCGGCGGGGGGGGGGGGCGGCGGGGGGGGGGGGTGTGTGTG	2.633
MLF1	NC_000003.12	13965	13988	23	GTGTTTGGGTCATGGGAGGGTGG	GTGTTTGGGTCATGGGAGGGTGG	1.391
MLF1	NC_000003.12	27114	27155	41	GGTGGGGGGACAGGAGGGAGGTGTGGGGGTTGGGGGTAAGG	GGTGGGGGGACAGGAGGGAGGTGTGGGGGTTGGGGGTAAGG	2.171
NR4A2	NC_000002.12	2261	2289	28	GAGTGGGATGAGTGGGGGGGTATGAGGG	GAGTGGGATGAGTGGGGGGGTATGAGGG	1.821
NR4A2	NC_000002.12	3558	3595	37	GGGCTGCGGCGGGGACTTCCGGGTGTCTGAGAAAGGG	GGGCTGCGGCGGGGACTTCCGGGTGTCTGAGAAAGGG	1.135
NR4A2	NC_000002.12	5312	5338	26	GGGGGTCAGGGAAGGGAAGGAGAGGG	GGGGGTCAGGGAAGGGAAGGAGAGGG	1.962
NR4A2	NC_000002.12	6006	6025	19	GGTAATAAGGGAGGGAGGG	GGTAATAAGGGAGGGAGGG	1.632

Sequence definition	NCBI Reference Sequence	Begining of G4 sequence (first nucleotide=0)	End of G4 sequence	Length	Given sequence (+ strand)	G4-forming sequence	G4HScore
NR4A2	NC_000002.12	6071	6098	27	GGGAGGAAGGGAAACCAGAGGGTGGGG	GGGAGGAAGGGAAACCAGAGGGTGGGG	1.63
PPP1R10	NC_000006.12	540	560	20	GGGAGGCCGAGGCGGGTGGG	GGGAGGCCGAGGCGGGTGGG	1.55
PPP1R10	NC_000006.12	1757	1775	18	GGGTGGGAGTGTAGTGGG	GGGTGGGAGTGTAGTGGG	1.667
PPP1R10	NC_000006.12	3449	3473	24	GGGGTGTGGGGGGGGGTTGCAGG	GGGGTGTGGGGGGGGGTTGCAGG	2.542
PPP1R10	NC_000006.12	4297	4320	23	GGGCGCGTTGGGCGGCGTCCGGG	GGGCGCGTTGGGCGGCGTCCGGG	1.13
PPP1R10	NC_000006.12	4431	4462	31	GGATCTGGGAAGCGGGATAGGGATGGATGGG	GGATCTGGGAAGCGGGATAGGGATGGATGGG	1.387
PPP1R10	NC_000006.12	4974	4995	21	GGAG4681TTGGGTGGGGGGG	GGAG4681TTGGGTGGGGGGG	2
PPP1R10	NC_000006.12	11858	11878	20	GGGAGGGAGGGACAGGTGTG	GGGAGGGAGGGACAGGTGTG	1.6
PPP1R10	NC_000006.12	19088	19112	24	GGGAAGCATGGGTGCTGGAAAGGG	GGGAAGCATGGGTGCTGGAAAGGG	1.292
WDR47	NC_000001.11	35	65	30	GGAGTCGCTGGGCCGGGAGGGGCGGACGTG	GGAGTCGCTGGGCCGGGAGGGGCGGACGTG	1.267
WDR47	NC_000001.11	401	445	44	GGGCTTGGGTGCCGACTGGGAGGGGGCTGGGGTCGTGGGTGAGG	GGGCTTGGGTGCCGACTGGGAGGGGGCTGGGGTCGTGGGTGAGG	1.636
WDR47	NC_000001.11	704	733	29	GGAGGAAGAGAGGGAGCGGCGGGGCTGGG	GGAGGAAGAGAGGGAGCGGCGGGGCTGGG	1.586
WDR47	NC_000001.11	776	806	30	GGGATGGGAGCAGGCCTTTGGGGATCTGGG	GGGATGGGAGCAGGCCTTTGGGGATCTGGG	1.4
WDR47	NC_000001.11	59383	59406	23	GGAGGGCTAAGGGATCTATGGGG	GGAGGGCTAAGGGATCTATGGGG	1.565
WDR47	NC_000001.11	5863	5887	24	GGAGGATGACGGGAGTGGGAAGGG	GGAGGATGACGGGAGTGGGAAGGG	1.5

Sequence definition	NCBI Reference Sequence	Begining of G4 sequence (first nucleotide=0)	End of G4 sequence	Length	Given sequence (+ strand)	G4-forming sequence	G4HScore
WDR47	NC_000001.11	61981	62015	34	GGGTGGGGTGGGAAGGGGTAGGGATGGATGGTTTAGGG	GGGTGGGGTGGGAAGGGGTAGGGATGGATGGTTTAGGG	2
WDR47	NC_000001.11	68784	68812	28	GGGAGATAGTGACTAGGGTGAATAGGGG	GGGAGATAGTGACTAGGGTGAATAGGGG	1.321

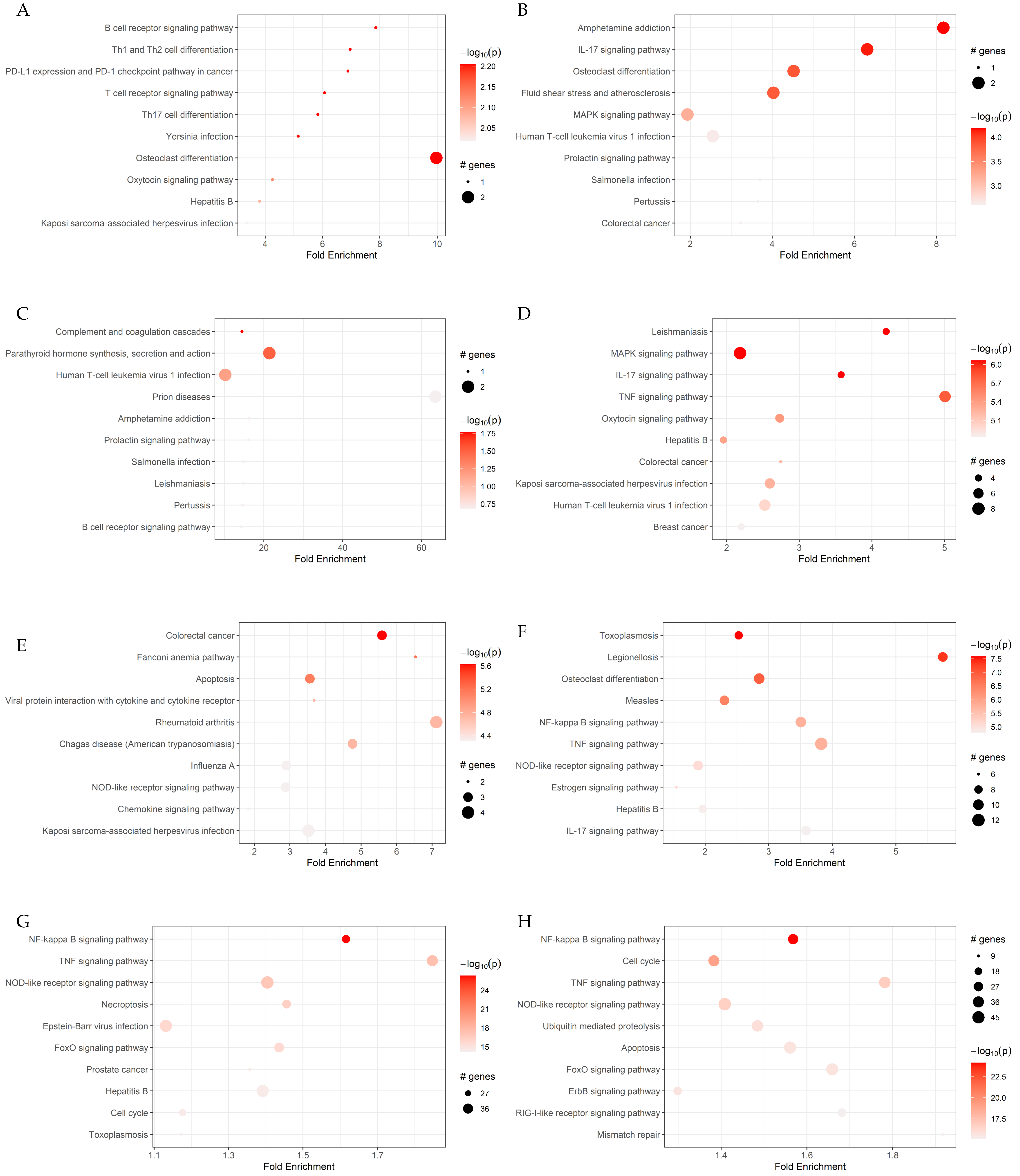

Figure S1. KEGG pathways of differentially expressed genes triggered by Na,K-ATPase inhibition by 3 μM ouabain (A, C, E, G) or K^+-free medium (B, D, F, H) in 0.5 h, 1 h, 2 h and 6 h, respectively.
